# Non-invasive endocrine monitoring indicates seasonal variations in gonadal hormone metabolites in dholes (*Cuon alpinus*)

**DOI:** 10.1093/conphys/cox001

**Published:** 2017-02-15

**Authors:** Jaruwan Khonmee, Suvichai Rojanasthien, Chatchote Thitaram, Jureerat Sumretprasong, Anurut Aunsusin, Chawin Chaisongkram, Nucharin Songsasen

**Affiliations:** 1 Department of Veterinary Bioscience and Veterinary Public Health, Faculty of Veterinary Medicine, Chiang Mai University, Chiang Mai 50100, Thailand; 2 Department of Food Animal Clinic, Faculty of Veterinary Medicine, Chiang Mai University, Chiang Mai 50100, Thailand; 3 Department of Companion Animal and Wildlife Clinic, Faculty of Veterinary Medicine, Chiang Mai University, Chiang Mai 50100, Thailand; 4 Chiang Mai Night Safari, Hang Dong, Chiang Mai 50230, Thailand; 5 Chiang Mai Zoo, Chiang Mai 50200, Thailand; 6 Center for Species Survival, Smithsonian Conservation Biology Institute, National Zoological Park, Front Royal, VA 22630, USA

**Keywords:** Dhole, gonadal steroids, non-invasive hormone monitoring, sexual behaviour

## Abstract

To date, there is no information on reproductive endocrinology of dholes (*Cuon alpinus*). The objectives of the present study were as follows: (i) to characterize longitudinal profiles of gonadal steroids; and (ii) to examine the relationship between gonadal hormones and sexual behaviours in dholes. Three breeding pairs and two bachelor males were included in the study. Among these, four animals (2 males and 2 females; 4 years old) were imported from The Netherlands to Thailand 3 months before the study onset; the remaining individuals (3 males and 1 female; 5–7 years old) were native born. Faecal samples were collected 3–7 days/week for 12 months, extracted and assessed for gonadal hormone metabolites using a validated enzyme immunoassay. Observations of behaviour were conducted in 30 min sessions, 3–5 days/week. For the three breeding males, testosterone was elevated (*P* < 0.05) from October to January in the two imported males, whereas the concentration of steroid metabolites was high from April to June and from September to November in the native male. However, there was no clear seasonal pattern of reproductive hormone in the bachelor group. Oestrogen metabolite level of imported females was elevated for 9–12 days in January, followed by a rise in progestagen concentration. For native females, oestrogen metabolites were above the basal values in April and September, each of which was followed by a rise in progestagen concentration that remained elevated for 77 and 112 days, respectively. Sexual behaviours, including solicitation, mounting and copulations, were observed during the oestrogen peak in all females. Our findings indicate that reproductive seasonality of dholes may depend on the animals’ origin and social group.

## Teaser

Non-invasive hormone monitoring was used to monitor reproductive hormone metabolites in dhole (*Cuon alpunus*) faecal samples. The findings indicate that dholes are seasonal breeders. However, the geographical origin of the animals may influence the onset of the breeding season. Finally, the study demonstrated evidence of polyoestrus in the female.

## Introduction

The dhole or Asiatic wild dog (*Cuon alpinus*) is a medium-sized, social canid. The species currently inhabits a fragmented range in southern China, India, Myanmar, Thailand, Laos, Vietnam, Malaysia and Indonesia (Durbin *et al*., 2004). Dholes are listed as ‘endangered’ by the International Union for Conservation of Nature (IUCN, 2016), mainly because of threats associated with habitat fragmentation, hunting, prey depletion, competition with other large carnivores (i.e. tigers and leopards) and diseases transmitted by feral/domestic dogs (Durbin *et al*., 2004). It has been estimated that there are <2500 mature individuals living the wild (IUCN, 2016). The dhole is secretive and lives in highly social, close-knit packs of three to 20 individuals, with a structure of rigid dominance hierarchies (Durbin *et al*., 2004). Dholes hunt co-operatively and maintain communication with pack members by whistling as they move through dense forests (Fox, 2004).

To date, there is limited information on dhole reproduction. Behavioural data gleaned from a 4 year study in India suggested that dholes are seasonal breeders (Paulraj *et al*., 1992). However, the time of breeding season varies among regions. Paulraj *et al*. (1992) reported that breeding occurs between August and December in India. However, a study conducted in the same country revealed that mating occurs between November and April, with peak activity observed during December and January (Durbin *et al*., 2004). Finally, dholes are thought to mate mainly during January and May in East Java (Durbin *et al*., 2004), and those housed in a zoo in Germany breed in January (Maisch, 2010). The oestrous period lasts 14–39 days, and sexual behaviours, including solicitation, copulatory tie and back-to-back posture have been observed during this period, as in other canids; the copulation period ranged from 1 to 9 days (Durbin *et al*., 2004). Based on behavioural observations, dholes exhibited seasonal polyoestrus with a period of 4–6 weeks between cycles (Durbin *et al*., 2004), which is different from other wild canids, such as the maned wolf (*Chrysocyon brachyurus*; Velloso *et al*., 1998; Songsasen *et al*., 2006), African wild dog (*Lycaon pictus*; Monfort *et al*., 1997) and red wolf (*Canis rufus*; Walker *et al*., 2002). The gestation period is ~9 weeks (Durbin *et al*., 2004; Fox, 2004), and litter size ranges from four to 12 pups (Durbin *et al*., 2004).

To date, there is no information on reproductive endocrinology of the dhole. Therefore, the main goal of the present study was to characterise longitudinal gonadal steroid profiles using a non-invasive endocrine monitoring technique developed for domestic and wildlife species (Schwarzenberger *et al*., 1996; Brown, 1995, 2011). The specific objectives were as follows: (i) to determine the influence of seasonality on gonadal hormone excretion; and (ii) to examine the relationship between reproductive hormones and sexual behaviours in females.

## Materials and methods

### Animals and collection of samples

A total of five males and three females maintained in two zoos in Chiang Mai, Thailand (18° 47′ 25″N, 98° 58′ 54″E) were included in the study. The three breeding pairs were housed at the Chiang Mai Night Safari, two (4 years old) of which were imported from The Netherlands 3 months before the study onset, and were kept off exhibit in a 3 m × 4 m (width × length) outdoor enclosure. During the study period, the two imported females gave birth; one had one pup that died soon after birth at the end of March and another had a litter of four pups at the beginning of April, one of which survived (hand rearing). The remaining pair (7 years old), born in Thailand were maintained on exhibit in a 5 m × 4 m (width × length) outdoor enclosure. The two bachelor males (5 years old) were born in Thailand and kept on exhibit 3 m × 4 m (width × length) at Chiang Mai Zoo. All animals received natural light and were fed fresh chicken once daily. Fresh faecal samples (~30 g) were collected 3–7 days/week for males and 5–7 days/week for females for 12 months. All samples were stored at −20°C until hormone extraction and analysis. All animal procedures were approved by the National Zoo's Institutional Animal Care and Use Committee.

### Faecal extraction

All chemicals were obtained from Sigma Chemical Company (St Louis, MO, USA), unless otherwise stated. Frozen faecal samples were thawed at room temperature, and 0.5 g of each well-mixed wet sample was placed in a glass tube containing 90% ethanol, vigorously shaken in a Multi Pulse vortexer (Glas-Col, Terre Haute, IN, USA) for 30 min, and centrifuged at 1300 g for 20 min. The supernatants were stored at −20°C until further analysis. Extraction efficiencies, determined by addition of 2500 d.p.m. ^3^H-steroid before extraction were 96.2% [coefficient of variance (CV) <10%] for testosterone (male samples) and 88.9% (CV <10%) for progesterone (female samples).

### Enzyme immunoassay

Testosterone, oestrogen and progestagen metabolites were quantified by enzyme immunoassay. Antibodies for testosterone (polyclonal testosterone R156/7; 1:8500 dilution), oestrogen (polyclonal estrone conjugate ECR522; 1:20 000 dilution) and progesterone (monoclonal pregnane CL425; 1:10 000 dilution) were obtained from the University of California, Davis, CA, USA. Cross-reactivities of R156/7 and ECR522 have previously been reported by Munro *et al*. (1991), whereas that of CL425 was reported by Graham *et al*. (2001).

Serial dilutions of pooled faecal extracts produced displacement curves parallel to those of the appropriate standards. Recovery of added standard to pooled faecal extracts demonstrated significant (*P* < 0.05) recovery (testosterone, *y* = 0.77*x* + 39.47, *R*^2^ = 0.997; oestrogen, *y* = 1.16*x* + 0.28, *R*^2^ = 0.998; and progestagen, *y* = 0.79*x* + 2.30, *R*^2^ = 0.998). Inter-assay CVs were <15% and intra-assay CVs <10%. Assay sensitivities were 2.3, 1.95 and 0.78 pg/well for testosterone, oestrogen and progestagen analyses, respectively.

### High-performance liquid chromatography

The quantities and relative proportions of immunoactive testosterone, estrogen and progestagen metabolites in dhole faecal extracts were determined using reverse-phase high-performance liquid chromatography (HPLC; Microsorb C-18 Column, Rainen Inc., Woburn, MA, USA). For each sex, five faecal extracts were combined, air dried, resuspended in 1 ml methanol, dried under air and stored at −20°C until analysis. Extract pools were reconstituted with 0.5 ml phosphate-buffered saline and filtered through a C-18 spice cartridge (VWR, West Chester, PA, USA), eluted with 5 ml methanol, air dried and spiked with known radio-labelled steroids (^3^H-testosterone, ^3^H-estradiol-17β, ^3^H-estrone, ^3^H-estrone-sulfate and ^3^H-progesterone, ~2500 d.p.m. each). Subsequently, the samples were dried down and resuspended in 300 µl methanol. Testosterone metabolites were separated using a gradient of 45% methanol over 90 min (1 ml/fraction; 1 ml/min flow rate). Oestrogen metabolites were separated using a 20–80% methanol gradient over 80 min, and progestagen metabolites were separated using 20–30–50–100% acetonitrile gradients over 15, 45 and 60 min, respectively. Co-elution profiles of the respective radio-labelled steroids in each HPLC run were determined by adding 100 µl of each HPLC fraction to 3 ml of scintillation fluid (Ultima Gold; Packard, Meriden, CT, USA) and counted in a dual-label channel β scintillation counter (Beckman, Fullerton, CA, USA). Each of the remaining HPLC fractions was then air dried, reconstituted in 0.2 ml assay buffer and quantified for immunoactivity by enzyme immunoassay.

### Behavioural observations

Quantitative behavioural data were collected 3–5 days/week in all breeding females during 30 min observation sessions conducted in the morning with instantaneous scan sampling (Martin and Bateson, 2007). During the observation period, all sexual behaviours, including solicitation, mounting and copulation, were recorded using an ethogram adapted from that developed for the maned wolf (Rodden *et al*., 1996). The behavioural data were then superimposed on endocrine profiles.

### Hormonal profiles and data analysis

#### Testosterone

Mean testosterone metabolites were calculated on a monthly basis and compared across months within each male to determine the influence of seasonality on hormone production using Kruskal–Wallis one-way analysis of variance (ANOVA) followed by Dunn's method (SigmaStat 3.0; SPSS Inc., Chicago, IL, USA). Significance was set at 95%.

#### Oestrogen and progestagen metabolites

Longitudinal profiles of oestrogen and progestagen metabolites were aligned to the day of the oestrogen peak (day 0). Day 0 was identified as the first day that oestrogen rose above baseline concentration by 2 SD (Songsasen *et al*., 2006). Baseline values for each individual were calculated by an iterative process, whereby high values (exceeding the mean plus 1.5 SD) were excluded. Each time the average was recalculated, and the elimination process repeated until no values exceeded the mean plus 1.5 SD. Data were reported as means ± SEM. Longitudinal steroid metabolite profiles were divided into three reproductive stages using modified criteria of those described by Velloso *et al*. (1998) and Walker *et al*. (2002), as follows: (i) anoestrus (days −30 to −11 before the oestrogen peak); (ii) pro-oestrus (days −10 to −1); (iii) oestrus (days 0–6); and (iv) dioestrus (days 7–93 or until the end of faecal collection in the two imported females). Differences in faecal steroid concentration among different stages of reproductive cycles (females) within the same individuals were determined by ANOVA followed by the Holm–Sidak method for multiple comparison. Differences were considered significant when the *P*-value was <0.05.

## Results

### High-performance liquid chromatography

**Figure 1:**
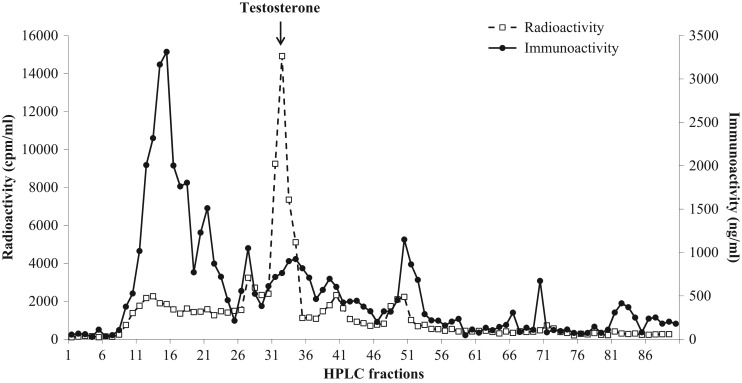
Co-chromatographic high-performance liquid chromatography separation of faecal testosterone metabolites of male dholes.

**Figure 2:**
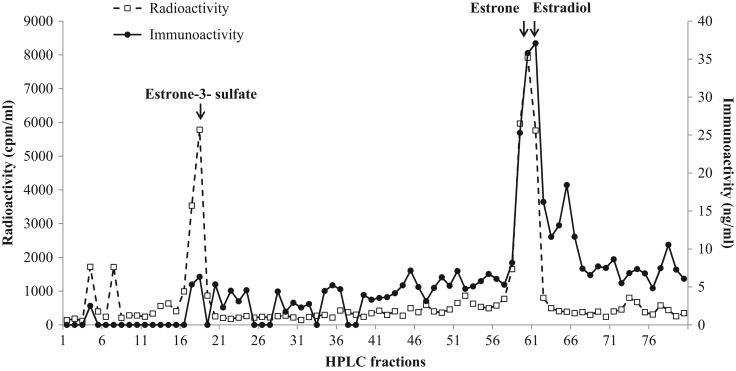
Co-chromatographic high-performance liquid chromatography separation of faecal oestrogen metabolites of female dholes.

**Figure 3:**
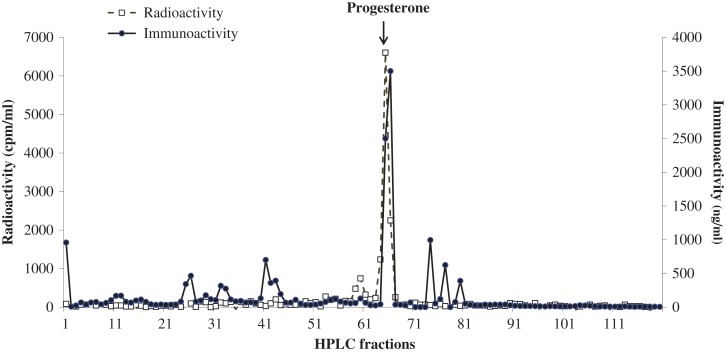
Co-chromatographic high-performance liquid chromatography separation of faecal progestagen metabolites of female dholes.

Evaluation of faecal extracts by HPLC revealed the presence of several testosterone metabolites, one (10%) of which exhibited similar retention time to testosterone (fractions 30–35; Fig. 1). The HPLC analysis of oestrogen metabolites demonstrated three immunoreactive peaks (fractions 15–20, 57–61 and 60–63), which co-eluted with estrone-3-sulfate, estrone and estradiol (Fig. 2). Progestagen immunoreactivity was associated with a single peak (fractions 63–68), which corresponded to the radio-labelled progesterone (Fig. 3).

### Steroid metabolite concentrations of male dholes

**Figure 4:**
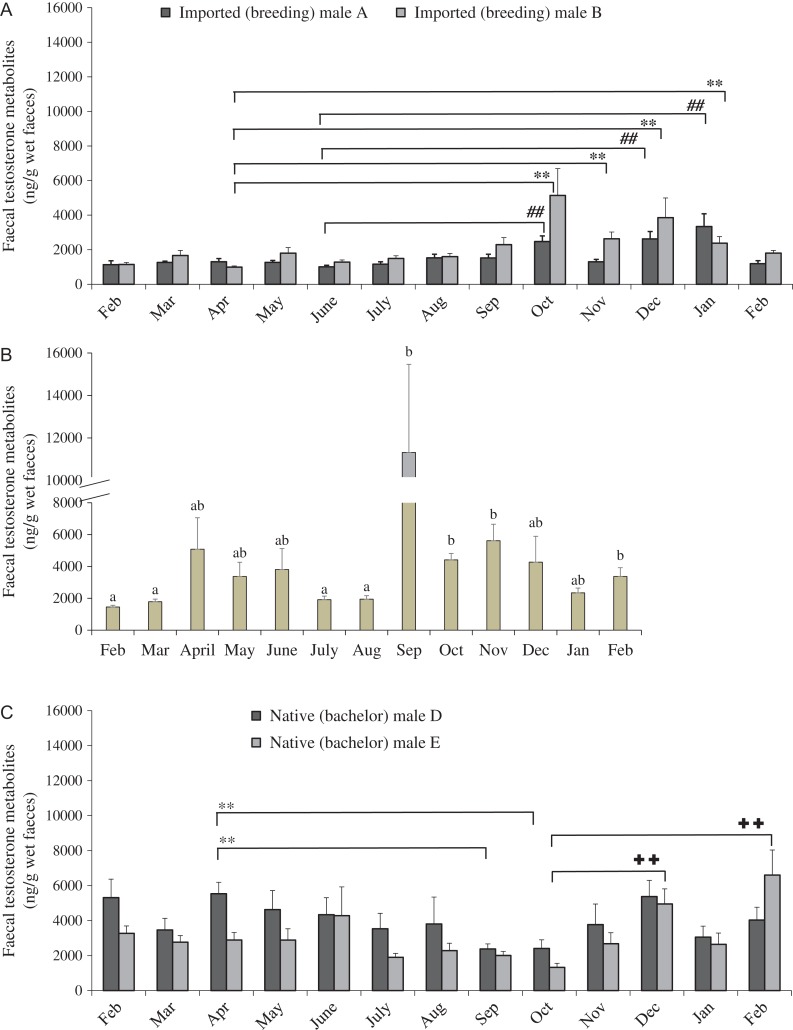
Mean ± SEM testosterone metabolite concentrations of two imported males housed in breeding pairs (**A**), a native male housed in a breeding pair (**B**) and two native males housed as a bachelor group (**C**). ^**, ##, ++^ Symbols or ^a,b^ superscript letters indicate significant differences among months within the same male. *P* < 0.05.

Mean ± SEM testosterone metabolites of imported (breeding), native (breeding) and native (bachelor) males are shown in Fig. 4A–C. For breeding males, testosterone concentrations varied among months. However, there were individual differences in the time during which high testosterone concentrations were observed. Specifically, testosterone metabolite concentrations of the two imported dholes (males A and B) were at low levels during February–September. Steroid concentrations significantly increased in October and remained elevated (*P* < 0.05) until January (Fig. 4A).

For the native male that was paired with a female (male C), testosterone metabolite concentrations were low in February, slightly elevated in April–June, significantly declined in July and August and then substantially increased in September and remained at high concentrations until November (*P* < 0.05; Fig. 4B). Seasonal variations in testosterone concentrations were not apparent in the two bachelor males (Fig. 4C).

### Steroid metabolite concentrations and reproductive behaviours of female dholes

**Figure 5:**
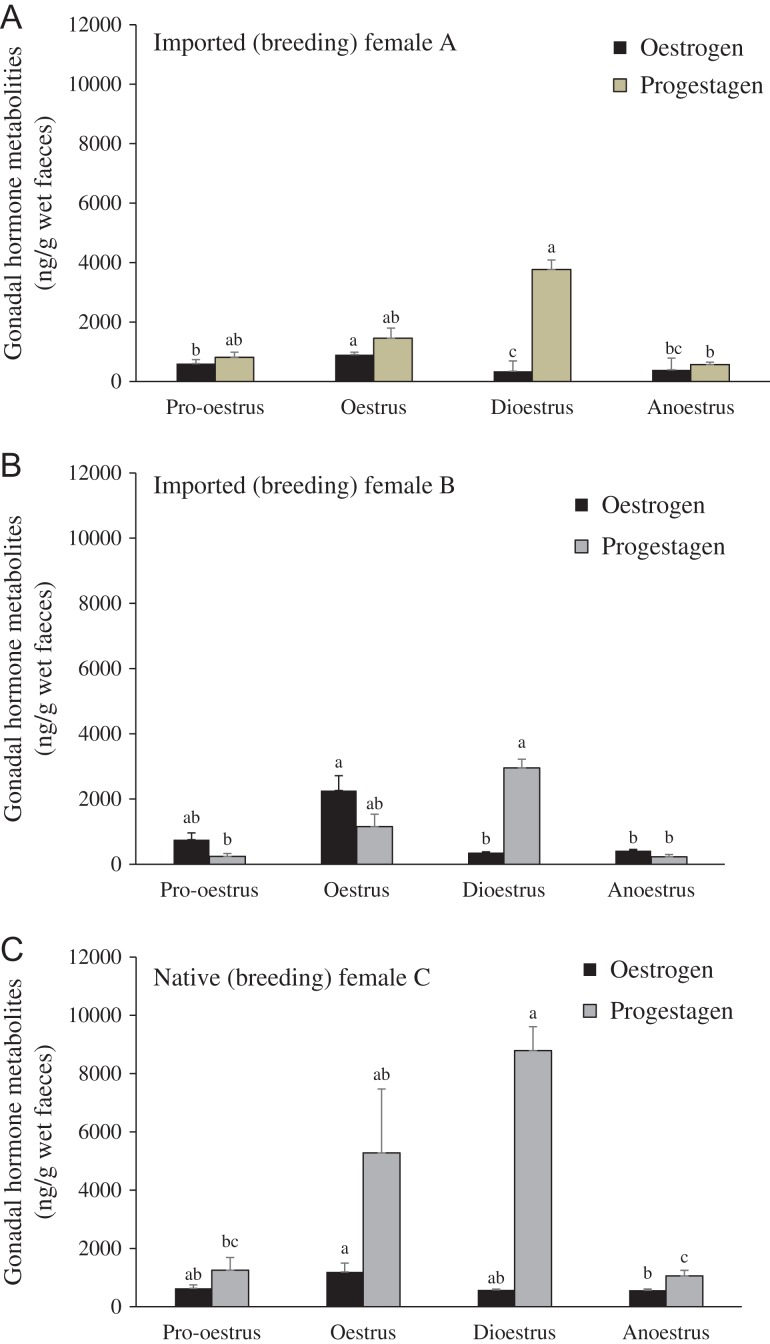
Mean ± SEM concentrations of ovarian steroid metabolites during pro-oestrus (days −10 to −1 of the oestrogen peak), oestrus (days 0–6), dioestrus (days 7–93) and anoestrus (days −30 to −11) of the two imported females (**A** and **B**) and the native female (**C**). ^abc^Different superscript letters indicate significant differences among reproductive cycles within the same individual for each steroid hormone (*P* < 0.05).

Faecal oestrogen and progestagen concentrations during various stages of the reproductive cycle are shown in Fig. 5. Regardless of the pregnancy outcome, all females exhibited a similar endocrine pattern. Specifically, mean oestrogen metabolite concentrations in all females were higher (*P* < 0.05) during oestrus than in pro-oestrus, dioestrus and anoestrus. Progestagen metabolite concentrations were at the baseline level during pro-oestrus, slightly increased during oestrus, significantly elevated during dioestrus (*P* < 0.05) and returned to the baseline levels during the anoestrous period.

**Figure 6:**
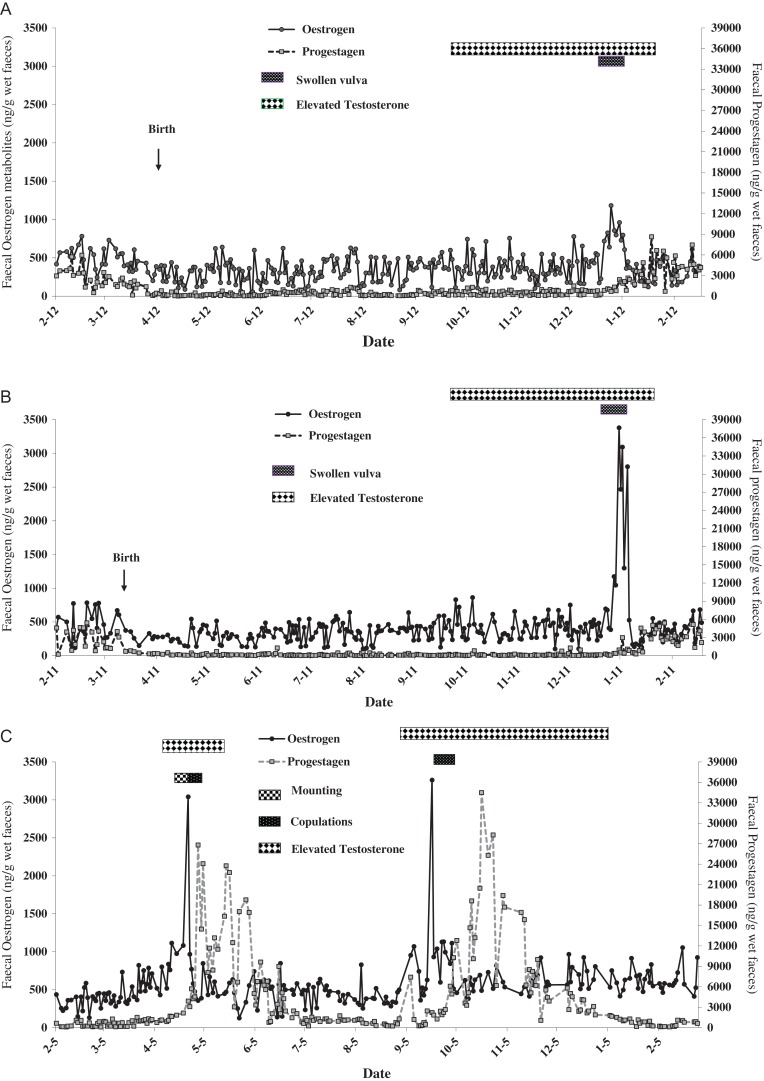
Longitudinal profile of gonadal steroids of the two imported females (**A** and **B**) and the native female (**C**) overlaid with reproductive events/behaviours and the interval during which elevated testosterone metabolites were observed in the respective male.

In all females, the increase in oestrogen coincided with vulval swelling and sexual behaviours, including solicitation, mounting and copulations (Fig. 6A–C). However, there were marked differences in reproductive season between imported and native females. The oestrogen peak of the imported females was observed in January, followed by a rise in progestagen concentrations, which remained elevated until the beginning of April (Fig. 6A and B). In the native pair, copulations were observed in April and September, but pups were not produced. Two oestrogen peaks, 149 days apart, were observed in the native female (in April and September); each was followed by increased progestagen metabolite concentrations, which remained elevated for 77 and 114 days, respectively (Fig. 6C).

## Discussion

Non-invasive faecal hormone metabolite monitoring has proved to be a valuable tool in advancing the understanding of reproductive functions and enhancing *ex situ* management of various wildlife species, including carnivores (Brown *et al*., 1995). The present study characterized gonadal hormone profiles in male and female dholes and investigated the influence of seasonality on the steroid excretion patterns in this species. Our data demonstrated that dholes are seasonal breeders, with gonadal hormone concentrations that varied among months, especially in breeding pairs. Specifically, in imported males, testosterone metabolites were elevated from October to January for the males, whereas steroid metabolites in the breeding native male were at a high level during April–June and September–November. The seasonal pattern of testosterone excretion corresponded to sexual behaviours and increased gonadal function in the females. Our findings also suggested that intrinsic (e.g. genetic) factors, rather than environmental conditions, may influence the time during which reproduction occurs. Finally, our data suggested that the dhole is a polyoestrous canid, a characteristic that is unique among species within the Canidae family.

In the present study, it was found that only a small proportion (10%) of androgen metabolites were excreted as testosterone in dhole faecal samples. This finding is similar to that reported in the raccoon dog (*Nyctereutes procyonoides*; Rudert *et al*., 2011). Given that the majority of metabolites were not associated with testosterone, this assay could not be used as an exclusive index for this steroid hormone. Nevertheless, variations in testosterone metabolite concentrations among seasons with high hormone concentrations observed during breeding periods in paired individuals indicated that this assay can be used to assess gonadal functions in male dholes. Unlike the African wild dog (Monfort *et al*., 1997), red wolf (Walker *et al*., 2002) and maned wolf (Velloso *et al*., 1998), which excrete mostly estradiol and estrone, dhole samples contained a significant amount of estrone-3-sulfate (20%) in addition to the two metabolites found in other canids. The immunoreactive progestagen peak of dhole samples was co-eluted with progesterone, which also differed from the findings in the African wild dog (Monfort *et al*., 1997), red wolf (Walker *et al*., 2002) and maned wolf (Velloso *et al*., 1998). In these three canids, progestagen metabolites excreted in faecal samples were less polar than progesterone. Given that increases in both oestrogen and progestagen concentrations coincided with reproductive behaviours observed in the three pairs, these assays can be used to evaluate reproductive functions in female dholes.

To date, information regarding dhole reproductive biology has been generated exclusively from behavioural observation of captive and wild individuals (Durbin *et al*., 2004; Maisch, 2010). It has been suggested that female dholes exhibit seasonal polyoestrus with 4–6 weeks between the end of the first and beginning of the next cycle (Durbin *et al*., 2004). In the present study, we observed that the non-pregnant female (female C) exhibited two estrous cycles during the study period, one from April to June and one from September to December, whereas the two pregnant females cycled only once a year. Although our observation was based on one female, it appeared that our endocrine data confirmed behavioural observations of others (Durbin *et al*., 2004), suggesting that dholes are unique amongst wild canids, which are seasonally monoestrous (Asa and Valdespino, 1998). Future studies that include multiple females are required to confirm this unique reproductive characteristic of the dhole.

Another interesting aspect is that there appeared to be differences in reproductive season between imported and native individuals, at least during the study period (1 year). In the imported males (males A and B), testosterone metabolites reached peak concentrations between October and January, which was consistent with the oestrous period observed in the females. Given that the two imported pairs gave birth in March and April, we estimated that breeding occurred in January, during the first reproductive season after they were transported to Thailand, and this pattern remained the same in the following year. This breeding period (January) differed from that observed in the native pair and wild counterparts living in Thailand (April and September). Specifically, the native pair exhibited breeding behaviours in April and September, which coincided with increased gonadal metabolite concentrations in both male and female. A camera-trap study conducted in eastern Thailand documented a photograph of two juveniles (~6 months old) in May, indicating that breeding probably occurred in September or October in wild individuals (Jenks *et al*., 2012). Furthermore, a lactating female was captured in a Thai protected area in mid-February, suggesting that this individual probably bred in late October to early November (N.S., personal observation). Thus, it appears that the reproductive season of imported dholes in the present study did not shift to the same period as that of native animals, even after being in Thailand for 1 year.

This is in contrast to observations in the maned wolf. The maned wolf breeding season differs by 6 months between the Northern and Southern hemispheres, with the peak breeding occurring in October (autumn) and April (autumn) in North and South America, respectively (Maia and Gouveia, 2002). Analysis of faecal testosterone concentrations of a male maned wolf transported from Brazil to the USA in June (i.e. the end of breeding season in Brazil) showed that this individual adapted to the reproductive season of North America shortly after being placed in the new environment, as steroid concentrations remained elevated until the following January (i.e. the end of the breeding season in the USA; N.S., unpublished data). Thus, it appears that reproductive seasonality in the maned wolf is regulated by environmental factors (photoperiod). The influence of photoperiod on reproduction has also been observed in the red wolf (*Canis rufus*; Walker *et al*., 2002), in which variation in faecal androgen metabolite concentrations is linked to changing day length; steroid concentration increases in late autumn (October) and peaks in winter (February). Given that imported dholes maintained the same reproductive seasonal pattern they had exhibited in Europe even after living in Thailand for 1 year, we suspect that the reproductive season of this species may be regulated by other factors, rather than the environment.

Based on morphology, geographical locations and genetic information, dholes are classified into 11 subspecies (Durbin *et al*., 2004; Iyengar *et al*., 2005). Recently, analysis of mitochondrial DNA of imported and native individuals included in the present study revealed that these two animal groups differed in the haplotype of mitochondrial control region (J. Kayman, personal communication). Specifically, the former was classified as having haplotype R, the type found mostly in captive dholes housed in European zoos. However, all native individuals were of haplotype U, a novel haplotype that has not been previously described (Iyengar *et al*., 2005). Thus, it is possible that the difference in seasonal cyclicity between imported and native dholes may be, in part, associated with genetic divergence. The influence of genetic factors on reproductive cyclicity has been previously shown in the red deer (*Cervus elaphus*; Asher *et al*., 2000).

By combining behavioural observation and endocrine monitoring, we have demonstrated that the dhole oestrous period ranges from 9 to 13 days, which is similar to that reported in other canids, including the grey wolf (*Canis lupus*; Seal *et al*., 1987), fennec fox (*Vulpes zerda*; Valdespino *et al*., 2002), red wolf (Walker *et al*., 2002), maned wolf (Songsasen *et al*., 2006) and domestic dog (Edqvist *et al*., 1975; Concannon *et al*., 1977; Wildt *et al*., 1979). Behavioural studies conducted on captive dholes reported that the oestrous period is 3–4 weeks, and the copulation period ranges from 1 to 9 days (Paulraj *et al*., 1992; Maisch, 2010). Courtship behaviours of dhole observed in the present study were similar to those reported in the previous study (Paulraj *et al*., 1992); these include mounting, copulatory tie and back-to-back copulatory posture. In the present study, two oestrogen peaks 149 days apart were observed in the native female; each was followed by increased progestagen metabolite concentrations that remained elevated for 77 and 114 days, respectively. Given that neither reproductive cycle resulted in pregnancy, the discrepancy in the length of the dioestrous period remains to be elucidated.

In summary, by using non-invasive hormone monitoring, we have established the first endocrine database characterising the reproductive cycle of the endangered dhole. Despite the small sample size, longitudinal faecal steroid monitoring through enzyme immunoassay assessments has revealed several reproductive characteristics of this understudied canid, including the evidence of the following: (i) seasonal polyoestrus in the females; and (ii) the potential of genetic impact on timing of reproductive seasonality. Future studies that include larger sample sizes and variation in pack composition may provide additional insights into reproductive mechanisms of this endangered canid.
